# Effectiveness of a multicomponent intervention to promote physical activity during the school day: rationale and methods of the MOVESCHOOL study

**DOI:** 10.3389/fpubh.2025.1565914

**Published:** 2025-03-12

**Authors:** Francisco J. Bandera-Campos, Alberto Grao-Cruces, Daniel Camiletti-Moirón, Fátima Martín-Acosta, Raúl Muñoz-González, María González-Pérez, Abel Ruiz-Hermosa, Miguel Vaquero-Solís, Carmen Padilla-Moledo, David Sánchez-Oliva

**Affiliations:** ^1^GALENO Research Group, Department of Physical Education, Faculty of Education Sciences, University of Cadiz, Puerto Real, Spain; ^2^Instituto de Investigación e Innovación Biomédica de Cádiz (INiBICA), Cadiz, Spain; ^3^ACAFYDE Research Group, Department of Didactics of Musical, Plastic and Body Expression, Faculty of Sports Sciences, University of Extremadura, Caceres, Spain; ^4^Social and Health Care Research Center, University of Castilla-La Mancha, Cuenca, Spain

**Keywords:** school-based intervention, physically active lessons, active breaks, active recess, adolescents

## Abstract

**Background:**

Increasing levels of physical activity (PA) and reducing sedentary time among adolescents during the school day is a pressing need. Emerging methodologies and strategies been shown to be effective in increasing PA levels and providing additional benefits for students, such us physically active lessons (PAL), active breaks (AB) and active recesses (AR). However, evidence concerning adolescents remains limited. This manuscript presents the methods and rationale of the MOVESCHOOL study, which aims was to examine the effects of a multicomponent school-based intervention during the school day on indicators of PA, sedentary time, health, executive functions and education in adolescents.

**Methods:**

A quasi-experimental study was conducted with the aim to involve a total of 800 students aged 12–14 years old from 11 schools (7th and 8th grade) in south-western Spain, five schools forming the intervention group and six schools forming the control group. The evaluation included two independent measurements: pre-intervention and post-intervention. The intervention lasted 29 weeks and consisted of a multi-component programme including a weekly PAL, two 5 min daily AB, and a daily AR. Primary outcomes included accelerometer-based PA and sedentary time, health-related physical fitness, academic indicators, and executive functions. For statistical analyses, descriptive, correlational, regression, and repeated measures ANOVA analyses will be applied. Additionally, qualitative data were gathered through semi-structured individual interviews and focus groups, and information will be evaluated with thematic analysis.

**Discussion:**

The MOVESCHOOL study represents a pioneering effort in Spain, being the first of its kind to evaluate the effectiveness of a multicomponent programme in secondary schools. Furthermore, this project provides valuable insights into the effects of a multicomponent school-based PA intervention on PA levels, sedentary time, health-related, cognitive, academic indicators and psychological health markers in secondary school students. The results of this study will make a significant contribution to the educational community, providing them with innovative teaching methods and strategies that have the potential to increase PA levels during the school day. In addition, this research promises to provide a transformative experience for educators, equipping them with tools to promote the holistic development of their students, enriching their academic performance and enhancing their well-being.

**Clinical trial registration:**

ClinicalTrials.gov, identifier NCT06254638.

## 1 Introduction

Increasing levels of physical activity (PA) and reducing sedentary time among children and adolescents is of paramount importance, as it helps to address the problems associated with current and lifelong physical inactivity, such as obesity and overweight (1–3). Additionally, it can positively affect other important factors during this stage, such as cognition and academic performance (4). Currently, PA levels among school-aged children are concerning. A systematic review in participants aged 5 to 18 years showed that children (≤12 years) spent 41 to 51% of the after-school period in sedentary time, whereas adolescents (>12 years) spent 57% of the after-school period in sedentary time (5). In addition, the school day is usually sedentary and Grao-Cruces et al. (6) found that Spanish secondary school students spent an average of 78% of the school day in sedentary behaviour.

Of all the contexts in which PA can be promoted at these ages, educational institutions have been identified as the most appropriate setting for intervention (7, 8). This is based on the fact that all children and adolescents have to spend a significant part of their waking hours at school, during crucial periods for the acquisition and consolidation of life habits. The presence of university-trained professionals in various fields and greater access to families also facilitates the role of these institutions as health promoters. In line with this, Spanish education legislation stipulates that schools should promote PA during the school day and encourage healthy lifestyles among their students (9).

Recently, World Health Organization (WHO) (8) published a policy brief toolkit describing the importance of integrating PA into schools. Among other things, to ensure that children aged 5 to 17 meet the recommendation of achieving 60 min of moderate to vigorous PA (MVPA) per day. In addition, the Sedentary Behaviour Research Network (SBRN) described the international evidence-based recommendations for school-related sedentary behaviour for youth (10), recommending that long periods of sedentary behaviour should be interrupted, that different types of PA should be incorporated, and that sedentary and screen-based learning activities should be replaced by active and non-screen-based learning activities. Therefore, in light of the recommendations from WHO and SBRN, there is a need to establish “active schools.”

Over the past decade, there has been a growing interest in developing viable strategies to increase PA levels throughout the school day without reducing time for other subjects. In this context, the scientific literature highlights the following strategies: physically active lessons (PAL), active breaks (AB), and active recess (AR). PALs are academic lessons other than PE (e.g., maths) that incorporate PA during teaching-learning activities without reducing the time devoted to the content of those subjects (11). These are based on integrating movement through exercises and games into areas other than PE to teach new academic content without compromising educational time (12, 13). A systematic review by Daly-Smith et al. (14) found that interventions combining PA with academic content showed higher levels of PA, time on task and academic outcomes, although this is less well established for adolescents. However, the evidence is not clear regarding some variables, such as changes in health-related physical fitness (HRPF) (13, 15, 16) or cognitive functions (13), as there are studies reporting significant effects and others that do not. Moreover, this lack of clarity is exacerbated in secondary education, where the existing evidence is more limited (13, 14). Therefore, further research in this area is necessary.

AB involves short bouts of PA, often of MVPA intensity, delivered by teachers during or between curricular lessons (12). Previous studies (12, 14, 17) have shown that AB significantly improves PA levels, but the results are inconclusive regarding the benefits on cognitive function and classroom behaviour. Once again, few studies have evaluated the effectiveness of AB in secondary education (14, 17). Specifically, at this stage, it has been demonstrated that 4 min AB are sufficient to achieve significant improvements in cognition (18). Although the duration of AB (ranging from 4 to 10 min) was consistent across studies, variations were observed in the frequency of application [ranging from two (19) to four (18) per school day].

Recess during the school day can be defined as “the non-curricular time scheduled between lessons” (20), and usually involves access to outdoor spaces and provides students with opportunity for unstructured PA and to socialize with their peers (21). Recess is a time with potential to contribute up to 40% of the daily MVPA recommendations (22). Therefore, AR can be described as recess that provides opportunities for students to engage in PA during school day (23). Previous studies (24, 25) reported that PA during recess can lead to improvements in academic, cognitive, behavioral, and emotional indicators.

This project was based on the framework of the Creating Active Schools (CAS) model (26). Based on the Behaviour Change Wheel (BCW) (27), the CAS models identified three sources of behaviour that are necessary and sufficient for the performance of a given volitional behaviour: Capability, Motivation and Opportunity. Furthermore, this model classified several intervention functions that could enable interventions (i.e., education, persuasion, empowerment, training, modeling, or constraints), as well as several target groups to develop the intervention. In this way, the CAS model framework identifies the multiple components necessary to establish schools as adaptive complex subsystems, which in turn facilitate the implementation of PA throughout the school. The lower half of the framework describes the internal factors of the school, while the upper half identifies factors related to teacher training, behavioral science, and the role of national and international organisations and policy development—the wider system beyond the individual school. In our project we focused on: (i) school leaders, who are responsible for leading the development of policy and vision statements and managing related resources; (ii) teachers, who are central to creating a positive social and physical environment, as well as implementing initiatives to promote healthy lifestyles; and (iii) students, who may form student councils or lead opportunities to adopt healthy lifestyles. By intervening with these groups, strategies to increase PA levels during the school day were developed and implemented through three opportunities (i.e., PAL, AB and AR).

Analyzing previous studies on interventions for promoting PA in schoolchildren (28–32), it is observed that a large part of these implement a single PA component (e.g., only PAL). Most single-component interventions in schools have shown limited or insignificant post-intervention effects (31, 32). Additionally, the possible low quality of existing systematic reviews, meta-analyses, or the studies included in them limits the ability to draw firm conclusions (32). Despite the limited evidence, multicomponent school-based interventions (targeting two or more components, such as PAL, AB, active commuting, etc.) have been proposed as one of the most promising approaches to promote PA in schools (32–35). However, systematic and meta-analyses examining the effects of multicomponent interventions show inconclusive results and generally small effects (36, 37). Evidence for these types of interventions on variables such as executive functions or academic performance is weak.

On the other hand, most of these multicomponent interventions have been carried out in primary schools (32), and few in secondary stage, with inconclusive effects on PA levels (36, 37). Therefore, there is a need to conduct studies that focus on the secondary level and test multicomponent interventions, as they promise greater effects on PA levels, and all that this entails.

Starting from the fact that this work is a protocol, its aim was to describe the methods and rationale of the MOVESCHOOL study, aimed to examine the effectiveness of a multicomponent school-based intervention on PA levels, sedentary time, HRPF, executive functions, academic indicators (school engagement, learning perception, academic performance and mathematical fluency), psychological health markers and motivational variables in adolescents. A secondary objective was to describe the methods used to evaluate the perception of school leaders, teachers and students (perceptions, limitations, strengths, areas for improvement, perceived benefits, enjoyment, motivation, training needs, fidelity and sustainability) about the intervention. Based on previous studies, this study hypothesized that participants in the experimental group, compared to those in the control group, would show higher PA levels during the school day, improved HRPF, executive functions, academic performance, psychological health, and motivation, as well as a reduction in sedentary time during the school day.

## 2 Methods and analysis

### 2.1 Study design

The MOVESCHOOL was a quasi-experimental study (trial registered on Clinicaltrials.gov: NCT06254638). This work was the protocol for the implementation of a school-based multicomponent intervention in the experimental group with pre- and post-intervention measures. MOVESCHOOL was a multi-center intervention whose management was designed to ensure effective collaboration and communication between the research groups. The research groups were composed of qualified researchers and graduates in PA and Sport Sciences from two universities [University of Extremadura (UEX) and University of Cadiz (UCA), Spain]. Both research groups followed a uniform study protocol for training, fieldwork, data collection and management, and quality control procedures. Continuous telematic communication was maintained throughout the study. A graphical summary of the study can be seen in the Supplementary file.

### 2.2 Participants and selection criteria

Participants in the MOVESCHOOL study were apparently healthy adolescents from secondary schools in the regions of Caceres and Cadiz (Spain). The MOVESCHOOL study established the following inclusion criteria for participants: (i) enrolled in 7th or 8th grade (12–14 years old); (ii) without any physical disabilities or health issues that could restrict PA levels; and (iii) having parental or legal guardian consent to participate in the study. Additionally, we included three criteria for schools: (i) having at least 60 students in 7th and 8th grade; (ii) not involved in any other PA or health promotion programs; and (iii) located within a 50-kilometre radius of the research group’s centers in Cadiz and Caceres.

**Figure 1:**
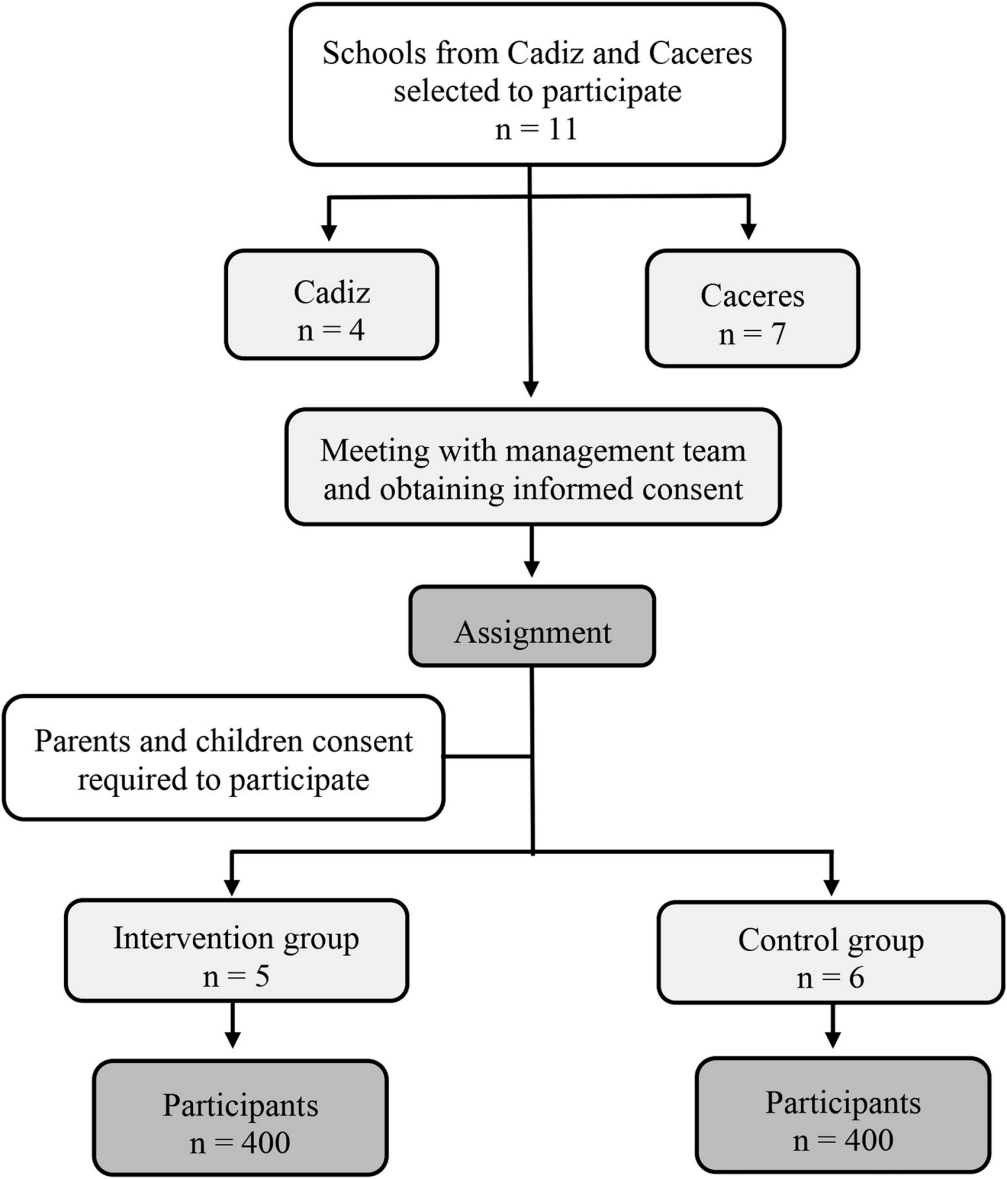
Flow diagram of study participants.

Figure 1 illustrates the flow diagram of the participants recruitment process. Seven schools in Caceres and four schools in Cadiz were selected to participate through a letter of invitation addressed to the school administration. A meeting was held with the school management teams of the selected schools, to explain the study and obtain their consent. Two study groups were established through a nonequivalent control group design: (i) intervention group and (ii) control group. In Cadiz, two intervention groups and two control groups were assigned; and in Caceres, three intervention groups and four control groups were assigned. Each group corresponds to a different school. All 7th and 8th grade students at the participating schools who provide their informed consent were invited to participate in the study. The parents of these students received an information document detailing the study, the inclusion criteria, the informed consent process, and an invitation to attend an information meeting at the school. For students to participate in the study, their parents or guardians had to sign the informed consent form along with the student’s consent.

The recruitment process was designed to achieve 800 participants (400 per group), which guarantees to detect small sized effects with an *α* error of 0.001 and a power (1 − *β*) of 0.95, even with an experimental dropout rate of 15% (GPower 3.1.9.4, Düsseldorf, Germany). To establish this sample size, Love et al. (38) were referenced, as they found an average effect size <0.1 for our main variable, MVPA time during the school day.

### 2.3 Interventions

**Figure 2:**
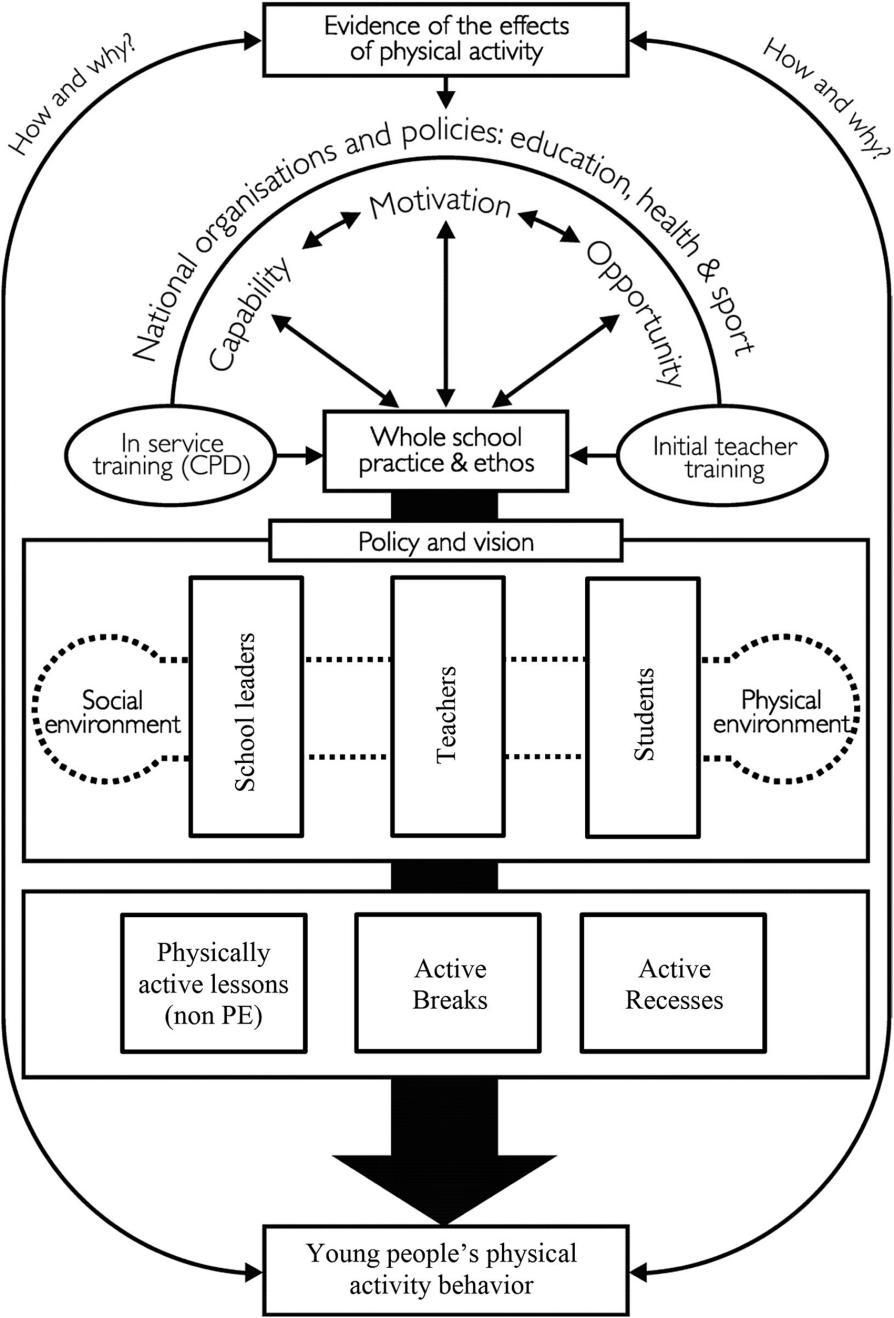
Creating active schools framework for MOVESCHOOL. Source: adapted from Daly-Smith et al. (26).

The MOVESCHOOL study implemented a multicomponent school-based intervention over a period of 29 weeks. As we have explained in the introduction section, the study is based on the CAS framework (26). In this way, and also following the COM-B behaviour change model, the three basic elements (i.e., Capability, Opportunity and Motivation) were worked on with teachers and school leaders in a kick-off meeting, where the protocol to be followed in the project was explained to all the teachers and the staff from each school before the start of the intervention. This ensured that each school had a whole-school overview and for the study to have a global perspective of each center. Similarly, we worked together with school leaders and teachers with the aim to increase students’ PA levels during the school day by implementing the three arms of the intervention: PAL, AB, and AR (see Figure 2).

#### 2.3.1 Physically active lessons component

The PAL intervention consisted of integrating PA into academic lessons in different subjects that were typically sedentary. One subject was selected for each week of the intervention. Out of the total number of lessons taught per week in the selected subjects, one lesson per week for 29 weeks was dedicated to the PAL intervention. Each of the PAL was developed outdoors during the intervention. The PAL sessions lasted 60 min and were fully designed with tasks that incorporated PA throughout the session. According to the results of a pilot study we conducted in a previous study (39), participants spent an average of 29.78% of the PAL session time in MVPA. Since the activities of PAL implemented in both studies was similar, we expect PA intensities also to be similar. The teacher assigned to this intervention group, who has been trained beforehand, supervises the implementation of the strategies in their academic lessons with the constant support of the research team. Before each PAL lesson, the research team and the teacher met to determine the specific content to be covered and to jointly develop the activities to be included in the lesson. Examples of PAL activities can be found in Grao-Cruces et al. (40) and here.

Teacher training was conducted in the month prior to the start of the intervention. It consisted of two 60 min workshops, led by the members of the research team, aimed at providing teachers with a foundation for implementing PAL in their teaching. The training covered: theoretical foundations of PAL, main characteristics of PAL, applicability and examples of PAL in secondary school subjects.

#### 2.3.2 Active break component

The AB intervention consisted of two daily ABs in a normal academic lesson. The timing of the AB was coordinated with the school management team, with one AB before recess and the other after recess. It was important that no AB are scheduled during PE classes or the lesson immediately following PE. This precaution ensured that the impact of the AB is not solely attributed to the PA that took place in the preceding PE lesson.

In order to facilitate the implementation of the AB, teachers used an adapted version of the “EUMOVE Active Breaks Platform,” a digital platform specifically designed for the implementation of AB (41). Therefore, before developing the intervention, the ABs were programmed in this platform. In this way, only the teachers who would be teaching the class at the time scheduled for the intervention were able to access the platform and start the programmed ABs. An example of an AB can be found here. This innovative tool made it possible to easily and quickly programme different types of AB, playing with variables such as PA duration or intensity. According to Daly-Smith et al. (14), each AB lasted 5 min and consisted of 4 different exercises, divided into 2 sets (4 rounds each) of 20 s of work and 10 s of rest. Finally, a cool-down consisting of deep breathing is performed. The activities selected for each AB included aerobic and strengthening activities (i.e., squats, lunges, jumps or skipping).

Teachers were given access to the platform and were tasked with delivering the AB according to the schedule provided by the research team. Throughout the intervention, students followed the instructions of an avatar who facilitates the session through the platform. To ensure optimal use of the platform, the research team provided extensive training and ongoing support to the teachers. During the initial phase of the intervention, the research team provided teachers with hands-on guidance for the first 2 weeks to ensure seamless integration of the AB and to address any queries or concerns that may arise. This support is designed to familiarise teachers with the platform and give them the confidence to deliver the AB.

#### 2.3.3 Active recess component

The aim of this component of the intervention was to increase levels of PA during school recess. To promote PA during school recesses, three strategies were developed: (i) environmental modification; (ii) free availability of sports equipment; and (iii) structured recess. Environmental modification provided a greater number of spaces where students could be active during playtime. The playground space was divided into zones and configured with different activities to vary space usage. The students were able to access to sports equipment in order to encourage the practice of PA during recess. Loose equipment had the advantage over fixed equipment or changes in infrastructure because it can be modified regularly. Structured recess includes organizing sports competitions and activities led by teachers. All of this is prepared by the research team together with the school’s PE teachers, and during its implementation, it was supervised by the technician along with the teachers assigned to recess duty.

#### 2.3.4 Control group

During the 29-weeks of intervention period, schools belonging to the control group received the standard academic lessons without methodological modification or inclusion of PA that could alter the usual levels of PA during school hours.

### 2.4 Implementation strategies

Several implementation strategies were used to ensure the success of this intervention programme. The primary approach was to assign a technician to each school in the experimental group. These technicians oversaw the day-to-day running of the project and maintained regular contact with the research groups and their counterparts in other schools. This process facilitated rapid decision-making on matters that required immediate attention or did not require a full meeting of the researchers. In addition, these researchers maintained ongoing communication with the teaching staff and school management teams at the experimental centers. Another implementation strategy involved providing training to teachers for the implementation of PAL, AB, and AR by the research team. The technicians assigned to each school were responsible for daily monitoring to ensure fidelity and/or guarantee the proper modification of any of the components in case they needed to be adapted. Fidelity was monitored through observation, direct questions to the teachers, automatic registration on the AB platform, and attendance lists for classes.

Finally, another important implementation strategy involved structured meetings at different stages of the intervention: (i) an initial meeting of the school management team with the research group to introduce the study and coordinate its development; (ii) an introductory meeting of the principal investigator with the participating teachers to present the project and coordinate its progress; (iii) regular meetings of the principal investigator with the participating teachers to evaluate and monitor the process; (iv) a final meeting of the school management team with the research group to conclude the project.

### 2.5 Measures

Participants were assessed at baseline (October 2023) and post-intervention (May 2024). Post-intervention measure of accelerometers-based variables were assessed during the last weeks of intervention, due to the characteristics of the indicators. To minimize variability in assessments, all measurements were conducted in schools by researchers who have received prior training.

#### 2.5.1 Primary outcome measures

##### 2.5.1.1 Physical activity and sedentary time

The Actigraph device (Actigraph GT3X+, Inc., Pensacola, FL, United States) was utilized for assessing PA at different intensities (light, moderate and vigorous) and sedentary time throughout the entire day. Participants were instructed to wear the monitor on their non-dominant wrist for eight consecutive days. Participants were advised to wear the accelerometer throughout the day (including sleeping time), except during aquatic activities or situations where exposure to water is possible. The screening and data collection procedures adhered to established protocols employed in previous research involving adolescents populations (42). Data will be extracted and analyzed using the GGIR package. Inclusion criteria for the analyses consist of: (i) a minimum of 3 valid weekdays of data and at least 1 valid weekend day of data; (ii) a minimum recording duration of 10 h per day (42, 43). The power analysis is performed based on MVPA during the school day, as it is the main variable of the study.

Additionally, to gather information on screen time throughout the day and the devices used, screen time was assessed using the specific items of the Youth Leisure-time Sedentary Behaviour Questionnaire (YLSBQ) (44). This questionnaire assessed the amount of recreational time per day spent on television, video games, computers, tablets, and smartphones, separately during weekdays and weekends. The student must choose whether they spend “0 min”, “30 min”, “1 h”, “2 h”, “3 h”, “4 h” or “5 h or more” per day on each of these behaviours.

#### 2.5.2 Secondary outcome measures

##### 2.5.2.1 Health-related physical fitness

To assess HRPF, the following tests belonging to the “ALPHA Fitness Test Battery” (45) were used:

###### 2.5.2.1.1 20-m shuttle run test

This test was used to assess cardiorespiratory fitness. Participants ran back and forth between marked lines on a track 20-m apart, matching the pace of an audio recording. The test started at 8.5 km/h and increased by 0.5 km/h per minute. It concluded when the participant stops due to fatigue or failed to maintain the required pace for two consecutive attempts. The participant’s score was based on the last stage completed.

###### 2.5.2.1.2 Hand grip test

This assessment was used to assess upper body maximal isometric muscular fitness. Participants were measured with a validated hand-held dynamometer with an adjustable grip (TKK 5101 Grip D; Takey, Tokyo, Japan). First, the dynamometer’s grip was adjusted to the size of the participant’s hand (46). Throughout the assessment, participants stood in an upright position and grasp the dynamometer with one hand. They incrementally applied pressure to the dynamometer until reaching maximum force, sustaining this pressure for a minimum of two seconds, all while ensuring stability in the elbow, arm, and trunk. The test was conducted twice, with participants alternating between hands. The highest score attained with each hand was documented in kilograms, and the average value between the two scores is computed and stored for subsequent analysis.

###### 2.5.2.1.3 Standing broad jump test

This test was used to lower body explosive muscular fitness. Participants positioned themselves behind a designated line with their feet shoulder-width apart. With a slight swing, they leapt forward as far as possible using both feet. If participants rested their hands on the ground or lift their feet upon landing, the test was considered invalid. This assessment was conducted twice, and the greatest distance achieved was measured in centimeters for analysis purposes.

###### 2.5.2.1.4 Body mass index and waist circumference

Both variables were used to assess body composition. Body mass index was calculated as weight divided by height squared (kg/m^2^). The weight measurement was taken using an electronic scale (type SECA 861; range, 0.05 to 130 kg; accuracy, 0.05 kg), and the height measurement was taken using a telescopic height instrument (type SECA 225; range, 60 to 200 cm; accuracy, 1 mm) taking into account the Frankfort plane. In both measurements participants were barefoot. Waist circumference was measured with a non-elastic tape (SECA 200; range, 0 to 150 cm; accuracy, 1 mm). The tape was positioned in the frontal plane at the midpoint between the superior iliac spine and the costal border at the mid-axillary line. The measurement was taken twice, and the mean of the two measurements was recorded. If there was a difference between measurements greater than 1 cm, a third measurement.

##### 2.5.2.2 Executive functions

The executive functions of inhibition, cognitive flexibility and working memory were assessed as main indicators of cognitive function. These were measured using the NIH Examiner programme (47) through the (i) Flanker task; (ii) Shifting task; and (iii) N-Back protocols, respectively.

###### 2.5.2.2.1 Flanker task

This assessment measured response inhibition and cognitive control (48). During the task, participants focus their attention on a central fish among a row of five displayed on the screen. The central fish is marked either above or below it. Each trial lasts for 1,000 milliseconds, during which participants are instructed to promptly indicate the direction the central fish is facing.

###### 2.5.2.2.2 Shifting task

This assessment measured cognitive flexibility (47). During the test, three figures of different colors are displayed on the screen, with one figure positioned at the top and one in each corner. The word “SHAPE” or “COLOR” appears on the screen, accompanied by auditory prompts from the computer. Participants are instructed to associate the top figure with one of the corner figures based on either its color or shape, depending on the prompt. If the word “COLOR” is heard, participants should select the corner figure with the same color as the top figure. Conversely, if the word “SHAPE” is heard, participants should select the corner figure with the same shape as the top figure.

###### 2.5.2.2.3 N-Back

This assessment measured working memory (49). During the test, participants are presented with a series of screens. The first screen displays a white square in a specific location, followed by a number that participants are instructed to read aloud. Subsequently, a second screen appears featuring a white square, which may be located in the same position as the previous square or in a different one. Participants are tasked with recalling the location of the preceding square.

##### 2.5.2.3 Academic indicators

###### 2.5.2.3.1 School engagement

To evaluate this variable, the Spanish version of the Utrecht Work Engagement Scale (UWES-S-9) was used (50). The UWES-S-9 is composed of 9 items that reflect the three dimensions of engagement: (i) vigor; (ii) absorption; and (iii) dedication, each dimension is represented by three items that are evaluated through a Likert-type scale, ranging from “never” (0 points) to “always” (6 points).

###### 2.5.2.3.2 Learning perception

Learning perception was assessed by the questionnaire developed by Abella et al. (51). The questionnaire consists of 8 items that measure two dimensions: “perceived learning” (items 1–4) and “satisfaction with learning” (items 5–8). Participants rate their agreement with each item on a five-point Likert scale, with “1” indicating “strongly disagree” and “5” indicating “strongly agree.”

###### 2.5.2.3.3 Academic performance

This variable was evaluated through the marks reported by the schools in the three official evaluations of the academic year, specifically in December, April and June. The evolution of students throughout the school year is analyzed based on their grades in all subjects.

###### 2.5.2.3.4 Mathematical fluency test

Immediately after the end of the cognition tests, a mathematical fluency test was carried out by means of test number 6 of the Woodcock protocol (52). In this test, participants were given a three-minute period to perform as many simple mathematical calculations as possible.

##### 2.5.2.4 Psychological health

Health status was assessed using the adapted version for children and adolescents of EuroQol five dimensions three level (EQ-5D-Y-3 L) questionnaire (53). This questionnaire evaluates health status across five dimensions: (i) mobility; (ii) self-care; (iii) usual activities; (iv) pain/discomfort and (v) anxiety/depression. Participants rate their level of difficulty or problems in each dimension using a Likert-type scale with three response options: (i) no problem, (ii) some problems, and (iii) many problems. Additionally, the questionnaire included a visual analog scale (VAS) to gauge general health, where participants assigned a score between 0 and 100 to indicate their current perception of overall health. Furthermore, self-perceived health was assessed using the classic self-reported health item (54). Participants categorize their health as either “excellent” (5); “very good” (4); “good” (3); “fair” (2); and “poor” (1).

##### 2.5.2.5 Motivational variables

###### 2.5.2.5.1 Novelty need satisfaction scale

It evaluated novelty (55). Five of the 19 questions that make up the original scale were selected. Participants were asked to rate their agreement with these five statements on a Likert-type scale, with following levels: (1) “strongly disagree”; (2) “disagree”; (3) “neither agree nor disagree”; (4) “agree”; and (5) “strongly agree.”

###### 2.5.2.5.2 The Spanish version of the sport satisfaction instrument

It evaluated enjoyment and boredom (56) in general studies. This scale consists of eight items measuring intrinsic satisfaction, with two subscales: satisfaction/enjoyment (five items) and boredom (three items). Participants rate their agreement with the items related to fun or boredom on a five-point Likert-type scale, with following levels: (1) “strongly disagree”; (2) “disagree”; (3) “neither agree nor disagree”; (4) “agree”; and (5) “strongly agree.”

###### 2.5.2.5.3 School climate

School climate was assessed by Students’ Perception of School Climate scale (PACE-33) (57). For this study, we used four indicators: “Student-teacher relationships” (4 items), “physical safety” (4 items), “group cohesion” (4 items) and “methodological resources” (3 items). Participants rate their agreement with each item on a five-point Likert scale, with following levels: (1) “strongly disagree”; (2) “disagree”; (3) “neither agree nor disagree”; (4) “agree”; and (5) “strongly agree.”

##### 2.5.2.6 School leaders’, teachers’ and students’ perception about the suitability and the implementation of PAL, AB, and AR

To gather qualitative information on the implementation of the multicomponent intervention and analyze the process, individual interviews were conducted with the participating teachers and school leaders. Additionally, focus groups with students were organized, adhering to the guidelines outlined in Finn and McInnis’s study (58). This way, useful information was obtained such as perceptions, limitations, strengths, areas for improvement, perceived benefits, enjoyment, motivation, training needs, fidelity and sustainability, from the perspective of the stakeholders who have been directly involved in the intervention. The interviews and focus groups were facilitated by the same researchers across all study provinces and were exclusively conducted in experimental centers. For the interviews, 1 school leader and a group of 4–5 teachers per center, who were involved in and familiar with the intervention, were selected. All interviews were conducted individually. These individual interviews were semi-structured, lasting approximately 30 min each, and were audio recorded for analysis. The focus groups, comprising 6–8 students selected (heterogeneous profiles), were also semi-structured and last around 30–40 min. Similar to the interviews, they were audio recorded to ensure accurate documentation of insights. The interviews were fully transcribed for the subsequent analysis of the data obtained from them.

#### 2.5.3 Confounding variables

##### 2.5.3.1 Sociodemographic characteristics

The socioeconomic status of participants was assessed using the Spanish-adapted version III of The Family Affluence Scale (FAS III) (59). This scale comprises six questions concerning the purchasing level of the participant’s family. Each question is rated on a categorical scale, and the total score, derived from the sum of responses across all six items, yields an aggregate index ranging from 0 to 13.

##### 2.5.3.2 Dietary patterns

Adherence to the Mediterranean diet was evaluated using the updated version 2.0 of the KIDMED questionnaire (60). The KIDMED 2.0 questionnaire comprises 16 questions that participants respond to. These questions encompass both positive and negative aspects related to the Mediterranean diet. Participants’ scores are calculated based on their responses, and the scores are categorized into three levels: (i) ≥ 8, indicating optimal adherence to the Mediterranean diet; (ii) = 4–7, suggesting a need for greater adherence to the Mediterranean diet; and (iii) ≤ 3, indicating low adherence to the Mediterranean diet.

### 2.6 Data analysis

Continuous variables will be presented as mean and standard deviation or median and interquartile range, as appropriate, while categorical variables will be expressed as frequency and percentage. A cross-sectional approach will be utilized, employing descriptive, correlational, regression, and differential analyses, along with Structural Equation Modeling. To evaluate the effects of the interventions on outcomes, repeated measures analysis will be conducted, with outcome measures serving as dependent variables in separate models, the intervention as an independent variable, and controlling for potential confounders (i.e., gender). Non-parametric analyses will be conducted if normality analyses indicate non-normal distributions. Quantitative analyses will be conducted using the SPSS v.29.0 statistical package (IBM, Armonk, NY, United States), with a confidence level of 95%.

Additionally, qualitative data from semi-structured interviews and focus groups will be analyzed using NVIVO software (61). Content analysis strategies will be employed, involving two phases: a deductive phase aimed at identifying information relevant to the study’s objectives, followed by an inductive phase focused on identifying teachers’ and students’ experiences, thoughts, and reflections related to AR, AB, and PAL (62).

To mitigate potential biases associated with the quasi-experimental design, we will apply statistical adjustments to control for confounding variables (i.e., gender, city, socioeconomic status) and ensure the robustness of our findings. Additionally, sensitivity analyses will be conducted to assess the impact of potential biases.

## 3 Discussion

This paper describes the protocol for a quasi-experimental study that aims to test the effectiveness of a multicomponent intervention based on the inclusion of PA, through PAL, AB and AR during school day, on PA levels, sedentary time, HRPF, executive functions, academic indicators, psychological health markers and motivational variables in secondary education students.

As noted above, there is a need for studies that implement interventions targeting adolescents, since this age group is less studied (32). Of the 23 reviews and/or meta-analyses presented, only 3 reviews focused specifically on secondary schools (students aged 12 to 18 years). Although 13 studies were conducted in primary and secondary schools together (aged 6 to 18 years), no conclusive results were found for secondary education. The systematic review of PALs by Norris et al. (13) found that of the 42 studies included, only 3 were conducted in secondary schools. The systematic review of ABs interventions by Daly-Smith et al. (14) included 8 studies, but only one involved secondary school students. Finally, Parrish et al. (22) also summarised ABs interventions and of the 42 studies included, 42 were conducted in primary schools and only one involved secondary schools.

In terms of intervention approach, Alalawi et al. (32) assert that the most promising interventions focused on PA integrated within the curriculum, and that some approaches, such as multicomponent interventions, appear more promising than others in fostering increased PA. Thus, based on the CAS framework (26) and working downwards to the practices and values of the whole school, an approach is created that encourages the involvement of relevant stakeholders (i.e., school leaders, teachers, pupils) and the creation of genuine physical and social environments that support PA. A paradigm shift is needed in secondary education, in line with the current Spanish education law (9), which emphasizes active methodologies, inclusion, and digitalization. This study supports these objectives by promoting physical activity through active methodologies. However, teacher training remains a critical area for improvement. Teachers and school leaders are key to the adaptive subsystem of the CAS framework, making initial and ongoing training essential to enhance their capability, motivation, and opportunities (27). Strengthening teacher training and engagement, as highlighted by Bernal et al. (35), is crucial for the sustainability and effectiveness of multicomponent interventions.

### 3.1 Strengths and limitations

The present study had several strengths that should be mentioned: (i) the study is carried out on a sample of secondary school, an educational stage that has received less attention in the scientific literature than primary and preschool education; (ii) this study implements a multicomponent intervention in the experimental group, allowing us to observe the effect of combining PAL, AB and AR. Most scientific evidence implements PAL, AB and AR separately; (iii) the PAL component uses a collaborative approach between professionals, teachers and researchers, which ensures greater sustainability of the intervention. This co-development process facilitates the integration of PA into the academic classroom and promotes long-term adoption by teachers; (iv) the study design will provide new evidence that improves the control of the internal validity of the results; (v) this study includes a mixed perspective, analyzing quantitative data but also including a qualitative way of exploring teachers’ and students’ perceptions of the intervention.

On the other hand, certain limitations should also be acknowledged: (i) the generalisability of the study findings may be limited to the specific school context and characteristics of the participants involved in the study (i.e., teachers, facilities, student motivation, age of the students, etc.); (ii) the sustainability and stability of the interventions may be compromised once the support of the research team is no longer available. AR are increasingly used, but sustaining the AB and PAL interventions in real school settings without the continuation of implementation strategies can be challenging; (iii) self-reporting of data depends on the accuracy and honesty of the participants’ responses, which may introduce biases in the results; (iv) teacher training that does not provide sufficient capability to become effective practitioners of the intervention throughout the school; (v) support from organisations and national policies for PA in schools and avoiding inadvertently promoting conflicting behaviours (e.g., sitting for long periods during lessons). While increasing levels of PA are currently recommended, it is necessary to adapt the pedagogical approach of schools and ensure that the training needs of key stakeholders are met.

## 4 Conclusion

The MOVESCHOOL study investigates the effectiveness of integrating PA into the school day through a multicomponent intervention including PAL, AB and AR. It will assess its impact on PA levels, sedentary time, HRPF, executive functions, academic indicators, psychological health and motivational variables. The MOVESCHOOL study is a valuable and innovative contribution to PA research in secondary education, particularly in Spain. It has the potential to inform educational policies and enhance student well-being. However, future research should consider long-term effects, broader implementation challenges, and additional psychological and social factors influencing PA engagement.

## Glossary

PA: Physical Activity
WHO: World Health Organization
SBRN: Sedentary Behaviour Research Network
PE: Physical Education
MVPA: Moderate to Vigorous Physical Activity
PAL: Physically Active Lessons
AB: Active Break
AR: Active Recess
CAS: Creating Active Schools
BCW: Behaviour Change Wheel
UCA: University of Cadiz
UEX: University of Extremadura
COM-B: Behaviour change model (Capability, Opportunity, and Motivation)
YLSBQ: Youth Leisure-time Sedentary Behaviour Questionnaire
HRPF: Health-related Physical Fitness
PF: physical fitness
EQ-5D-Y-3L: EuroQol Five Dimensions Three Levels
VAS: Visual Analog Scale
NNSS: Novelty Need satisfaction Scale
SSI: Sport Satisfaction Instrument
PACE-33: Perception of School Climate scale
FAS III: version III of the Family Affluence Scale
