# Supplementary material for: Effectiveness of a multicomponent intervention to promote physical activity during the school day: rationale and methods of the MOVESCHOOL study
Source: Front Public Health. 2025 Mar 12;13:1565914. doi: 10.3389/fpubh.2025.1565914 (PMC11936994; doi:10.3389/fpubh.2025.1565914)
Supplement: Supplementary file 1 [file Data_Sheet_1.pdf]

Effectiveness of a multicomponent intervention to promote physical activity levels during the school day: MOVESCHOOL study

Background

Increasing levels of physical activity (PA) and **reducing sedentary** time among adolescents during the school day is urgently needed as it will help to address the problems associated with physical inactivity (i.e. obesity and overweight).

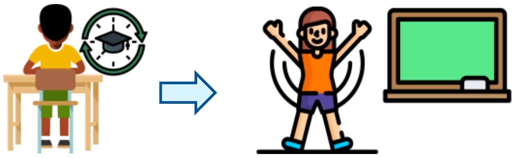

Risk factor: Spanish secondary school students spent an average of 81% of the school day engaged in sedentary behaviors.

Objective

Examine the effects → Multicomponent school-based intervention

During the school day:
- ACTIVE BREAKS
- PHYSICALLY ACTIVE LESSONS
- ACTIVE RECESS

On:
- PA
- Health
- Education
- Cognition

In adolescents

Methodology

Study Design

Quasi-experimental study

Participants

11 Schools
35 classes
840 students

- Spain: Caceres
- Spain: Cadiz

Intervention

Multi-component programme
29 weeks during academic year 2023/2024

1. **Physically Active Lessons** → One Physically Active Lesson per week
2. **Active Breaks** → Two Active Breaks of 5 minutes per day
3. **Active Recess** → An Active Recess per day

Measures

- ***Physical activity and sedentary time***: 7-days ActiGraph accelerometer. Sedentary time is also measured with YLSBQ (44).
- ***Health-related physical fitness***: ALPHA Fitness Test Battery (45): *Cardiorespiratory fitness:* 20-m shuttle run; *Strength*: hand dynamometer and standing long jump. Also, BMI and waist circumference.
- ***Cognition:*** the executive functions using the NIH Examiner programme (47): Flanker task, Shifting task and N-Back.
- ***Academic indicators:*** *School engagement:* UWES-S-9 (50); *Learning perception:* questionnaire developed by Abella et al (51); *Academic performance:* reported by the schools in the 3 official evaluations; *Mathematical fluency test*: test number 6 of the Woodcock protocol (52).
- ***Psychological health:*** *Health status:* EQ-5D-Y-3L questionnaire (53).
- ***Motivational variables:*** *Novelty:* NNSS (55); *Enjoyment and boredom:* the Spanish version of SSI (56); *School Climate:* PACE-33 (57).
- ***Teachers' and students' perception about the suitability and the development of multicomponent program:*** using individual interviews and focus groups.
- ***Dietary patterns:*** KIDMED 2.0 (60).
- ***Sociodemographic***: *sex, age, birth date, and socioeconomic status:* FAS III (59).

Design and procedure

Timeline: 2024 — Jun — Beginning AY 24/25 — Oct — 2025 — May-Jun

Work Plan

Study Coordination

Design Intervention → **School Recruitment** → **Initial Training** (Inform Consent) → **Baseline**

School-Based Intervention (29 weeks)

Baseline → **Experimental Group** (School-based Intervention) / **Control Group** (No Intervention) → **Post Intervention**

Quasi-experimental study

Impact

This study will provide:

- Valuable and innovative guidance for the education community to promote the adoption of methods and strategies to increase physical activity levels.
- A set of innovative resources that are freely available, evidence-based, easy to implement and context-appropriate.
